# A novel stent flow chamber system demonstrates reduced thrombogenicity of bioresorbable magnesium scaffolds

**DOI:** 10.1038/s41598-024-77266-0

**Published:** 2024-11-04

**Authors:** Monja Müller, Lars Ludwig, Hanna Englert, Katharina A. Riedl, May Cathleen Müller, Sandra A. Hemkemeyer, Manu Beerens, Reiner K. Mailer, Thomas Renné, Sabine Lang, Philine Baumann-Zumstein, Maike Frye

**Affiliations:** 1 Institute of Clinical Chemistry and Laboratory Medicine, University Medical Center Hamburg-Eppendorf, Hamburg, Germany; 2 German Centre of Cardiovascular Research (DZHK), Partner Site Hamburg, Luebeck, Kiel, Hamburg, Germany; 3 Department of Cardiology, University Heart and Vascular Center, University Medical Center Hamburg-Eppendorf, Hamburg, Germany; 4 Center for Interdisciplinary Cardiac Imaging, University Medical Center Hamburg-Eppendorf, Hamburg, Germany; 5 Center for Thrombosis and Hemostasis (CTH), Johannes Gutenberg University Medical Center, Mainz, Germany; 6 Irish Centre for Vascular Biology, School of Pharmacy and Biomolecular Sciences, Royal College of Surgeons in Ireland, Dublin, Ireland; 7 Biotronik AG, Bülach, Switzerland

**Keywords:** Coronary interventions, Stent flow chamber, Thrombogenicity, Stent, Bioresorbable magnesium scaffold, Platelets, Magmaris RMS, BIOmag, Freesolve RMS, Cardiac device therapy, Interventional cardiology, Preclinical research

## Abstract

Coronary artery disease (CAD) is characterized by narrowing and subsequent blockade of coronary arteries, and imposes a significant health and economic burden. Stent and scaffold devices are introduced in advanced CAD to improve vascular stability and restore blood flow. Although in vitro flow systems like the Chandler loop have been developed to enhance the understanding of interactions between device materials, their coatings, and vascular cells, imaging-based in vitro analysis of device performance is limited. In this study, we established a novel stent flow chamber system designed to assess the thrombogenicity of bioresorbable magnesium scaffold (RMS) and stent materials in vitro. Additionally, we compared the thrombogenicity – an important clinical parameter in stent performance – of the Magmaris-316 L stainless steel stent with its predecessors, Magmaris RMS and a prototype of the third-generation RMS (DREAMS 3G). Analysis of platelet adhesion and coverage of the different devices under flow conditions demonstrated that the Magmaris RMS exhibits reduced thrombogenicity compared to the Magmaris-316 L stainless steel stent. Moreover, thrombogenicity of the DREAMS 3G prototype, composed of BIOmag material, is further decreased compared to its predecessors. The observed reduction in thrombogenicity of the DREAMS 3G prototype in vitro suggests additional improvements in clinical safety and efficacy, highlighting its promise for treating CAD. Future research on this prototype may thus open avenues for analyzing other blood components and patient-derived endothelial cells. In line with the 3R principles, this approach may also help reduce the need for animal testing.

**Supplementary Information:**

The online version contains supplementary material available at 10.1038/s41598-024-77266-0.

## Introduction

Cardiovascular diseases remain a leading cause of morbidity and mortality worldwide, taking an estimated 17.9 million lives each year according to the World Health Organization^1^. Coronary artery disease (CAD), a condition defined by the narrowing of blood vessels in the heart due to plaque deposits, poses a major health and economic burden to the society. The standard of care for advanced CAD is the introduction of stents at the vascular stenosis site, aimed to support the mechanical stability of the impaired vessel and restore blood flow^2^. Stent technology and materials have become increasingly sophisticated over the years, leading to reduced thrombogenicity and improved biocompatibility, and allow for a safer and more efficacious treatment. Nonetheless, stent failure due to restenosis remains a major clinical problem in the treatment of CAD.

While in vivo analysis of device performance includes a multifactorial analysis, a controlled in vitro environment can be essential to investigate cell type-specific and flow rate-dependent changes. These controlled environments enable specific analysis of hemodynamics, biomechanics, or effects on selected blood components^3,4^, and can enhance our understanding of cellular mechanisms regulated by different device materials and coatings. However, current in vitro platforms, such as the Chandler loop or flow loop^5^, do not allow for direct high-resolution microscopic visualization of device-cell interactions, e.g., platelet adhesion and aggregation on the device surface.

Recent advances in the field of interventional cardiology include the development of bioresorbable scaffolds (BRSs). In contrast to permanent stent material, BRSs provide a temporary scaffold and are resorbed within 1–36 months, dependent on the backbone material^6^. This promising technology therefore has the potential to overcome many of the safety concerns associated with permanent stent material, such as hypersensitivity reactions, a delayed onset of neointimal growth, neo-atherosclerosis, and an increased risk of late or very late stent thrombosis^6^. Although bioresorbable magnesium scaffolds (RMSs) such as the Magmaris scaffold show particularly very low thrombosis in patients^7,8^, a direct and standardized comparison between RMSs remains difficult up to date.

Here, we compare a Magmaris-316 L stainless steel stent (backbone design equivalent) with Magmaris RMS^9,10^, and a prototype of the third-generation RMS scaffold (DREAMS 3G) manufactured by Biotronik, Bülach, Switzerland.

Using a novel stent flow chamber system, we confirm that Magmaris RMS showed reduced thrombogenicity in comparison to Magmaris-316 L^10^. Furthermore, our findings indicate that the thrombogenicity of the novel DREAMS 3G scaffold was even further reduced, implying the sustained and potentially improved clinical safety and efficacy for therapeutic applications. Our findings provide valuable insights into the intricate dynamics of platelet interactions with different device material and could provide the foundation for further in vitro assessments of RMSs and their clinical applications.

## Methods

### Acquisition of human buffy coats and ethics statement.

 Buffy coats from healthy donors were obtained from voluntary blood donations at the Institute of Transfusion Medicine at the University Medical Center Hamburg-Eppendorf, Germany. Written informed consent was obtained from each patient included in the study. The study protocol conforms to the ethical guidelines of the 1975 Declaration of Helsinki and the regional ethics committee approved the study (PV6039). For some buffy coats the requirement for informed consent was waived by the Central Ethics Committee, Hamburg, Germany given the discarded nature of the blood samples.

### Preparation of platelet-rich plasma from human buffy coats.

 Blood bags containing about 60 ml of buffy coat from citrated whole blood were provided as described above. To obtain platelet-rich plasma (PRP) pooled buffy coats were centrifuged at 800xg for 5min at room temperature (RT) with minimal acceleration and deceleration to separate remaining leukocytes and erythrocytes. Platelet counts were determined using the Siemens Healthineers Advia 2100 system according to manufacturer’s instructions. PRP was adjusted with autologous platelet-free plasma (PFP) to 3-4 × 10^5^ platelets/µl.

### Coronary scaffolds and stents.

**Figure 1:**
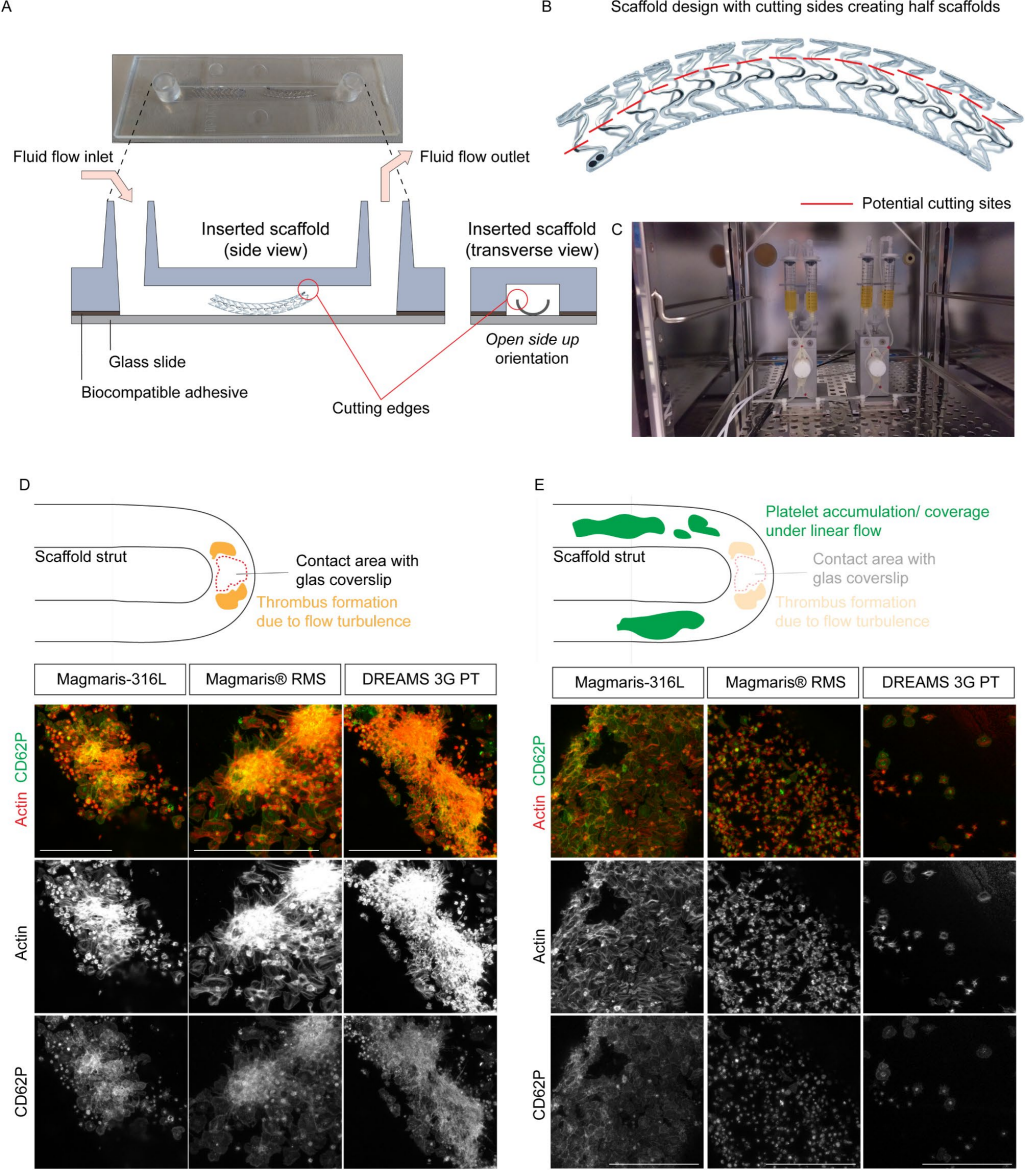
A novel in vitro flow chamber system to study thrombogenicity of scaffold material. (**A**) Schematic of scaffold embedding in side and front view. The *open side up* orientation of the half scaffold (with the cutting ends pointing upwards) in the channel slide was selected. (**B**) Cutting template of the stent and scaffold design to generate half stents and scaffolds using the indicated cutting edges. The stability of the scaffold is maintained via its connectors. (**C**) The scaffold embedded in the flow chamber is attached to the ibidi pump system, which is set up following manufacturer’s instructions. During the experiment fluidic units are placed inside a temperature-controlled CO_2_-incubator. (**D**) Micro-thrombotic events occurred at all scaffolds in the area of direct contact between the strut and glass coverslip. Individual flow experiments were conducted with PRP obtained from the same buffy coat for all scaffolds. Laminar shear stress of 10dyn/cm^2^ was applied for 2 h. Representative immunofluorescence of platelets attached to Magmaris-316 L, Magmaris and DREAMS 3G prototype (PT) scaffolds using an antibody against CD62P (green) and phalloidin (red, actin). Single channel images are depicted in grey. (**E**) Platelet accumulation and coverage of different scaffold materials can be reliably analyzed along the strut length under laminar flow. Individual flow experiments were conducted with PRP obtained from the same buffy coat for all scaffolds. Laminar shear stress of 10dyn/cm^2^ was applied for 2 h. Representative immunofluorescence of platelets attached to Magmaris-316 L, Magmaris and DREAMS 3G prototype scaffolds using an antibody against CD62P (green) and phalloidin (red, actin). Single channel images are depicted in grey. Scale bars: 50 μm (**D**,** E**).

Two RMSs, DREAMS 3G^9^prototype and Magmaris RMS (DREAMS 2G)^9,10^, and a stainless-steel Magmaris-316 L (backbone design equivalent) were compared in this study (all from Biotronik AG, Switzerland). The DREAMS 3G prototype used in this study, has the identical scaffold design as the Magmaris RMS and Magmaris-316 L stents but is made from BIOmag material^11^. Therefore, a head-to-head comparison of those three devices that share an identical design (Fig. 1B) with no variations in strut thickness (all devices: 150 μm), width (all devices: 150 μm) and cell compartment size allows for specific analysis of the thrombogenic potential of the device materials. Device backbones are coated with biodegradable poly-L-lactide (PLLA) that is loaded with sirolimus as an immunosuppressive and anti-proliferative drug, which elutes 69.4% within 90 days after implantation^9^. The DREAMS 3G with the final design will be commercially available under the name Freesolve.

### Device inflation and separation.

 Scaffold and stent devices were inflated under semi-sterile conditions using the Encore 26 Inflation device (Boston Scientific, MA, USA) according to the manufacturer’s protocol. Device backbones are designed with six crowns in each row and all adjacent rows are interconnected with two axial connectors. Devices were cut in half along a pre-defined area using a dissection microscope and sterile fine dissection instrument. With two rows of connectors per half device remaining, scaffold and stent stability was not compromised.

### Insertion of devices into channel slides.

 The embedding procedure into the sticky µ-Slide I Luer channel (channel height 0.8 mm, cat#80198, ibidi GmbH, Germany) was performed under semi-sterile conditions. The half scaffold or stent was oriented open-side-up with cut ends pointing upwards and the intact scaffold half pointing towards the sealing glass coverslip (cat#10814, ibidi GmbH, Germany). The glass coverslip was attached to the sticky slide including the embedded device and moderate pressure was applied to fully fit the device into the channel and to seal the slide airtight. All slides were additionally sealed with super glue to prevent PRP leakage and loss of vacuum.

### ibidi flow chamber system.

 The ibidi pump system (cat# 10902, ibidi GmbH, Germany) and the ibidi PumpControl software were employed to subject half scaffolds to controlled flow conditions. During the experiment fluidic units were placed inside a conventional cell culture incubator at 37 °C in a humidified atmosphere with 5% CO_2_. 13 ml of PRP were added to the reservoirs and the menisci of both sides were equilibrated. 10 ml fluid reservoirs, perfusion set (cat#10962, ibidi GmbH, Germany) and the channel slide were mounted according to the manufacturer’s protocol. Air bubbles were removed using a pre-defined ibidi PumpControl program and then arterial-like^12^ laminar flow (shear stress: 10dyn/cm^2^, plasma viscosity at 37 °C around 1.2–1.3 cPoise^13^ (0.012–0.013 dyn*s/cm^2^)) was applied for 2 h allowing platelets to recirculate.

Precise shear stress values have been determined by ibidi GmbH, Germany (https://ibidi.com/flow-accessories/118-perfusion-set.html) based on perfusion set and channel specific parameters, such as tube lengths and diameter, channel diameters, and work volumes.

### Immunofluorescence staining of platelet adhesion.

 After laminar flow exposure, slides were detached from the flow unit and washed twice with phosphate-buffered saline (PBS). Adherent platelets were fixed with 4% (w/v) paraformaldehyde for 45 min at RT. Cells were permeabilized with 0.5% (v/v) Triton-X-100 solution at RT for 5 min. Unspecific binding sites were blocked with 2% (w/v) BSA plus 0.3% (v/v) Triton-X-100 in PBS for 1 h at RT. Immunostaining was performed using a mouse anti-CD62P antibody (1:100, cat# ab6632, Abcam, UK) for 1 h at RT. After washing three times with 0.3% (v/v) Triton-X-100 in PBS, cells were incubated with fluorophore-labeled AffiniPure Fab Fragment anti-mouse 488 (cat# 115-547-185, Jackson ImmunoResearch, UK), and Alexa Fluor 594 Phalloidin (cat# A12381, Invitrogen, Germany) for 45 min at RT. Half scaffolds and stents were covered with PBS and z-stack images were taken within 2–3 days after conducting the experiment using a Leica SP8 confocal microscope with a HC PL APO 63x/1.4 Oil CS2 objective and the Leica LAS X SP8 software.

### Image quantification.

Quantifications were done using Fiji ImageJ. Actin pixel intensities (integrated density) were measured from maximum intensity projection images of acquired Z-stacks. To quantify category 2 platelets, spreading platelets were manually counted from 90 μm x 90 μm confocal z-stack images (8100 μm^2^).

As micro-thrombotic events were observed in all half devices within the region of direct contact between the strut and the glass coverslip, we selectively quantified device-blood component interactions along the strut length, while avoiding areas of artificially perturbed flow. Therefore, quantifications were not performed in a total blinded manner. Live videos of platelet flow were taken using a DS-Fi3 camera on the Nikon Eclipse Ts2R.

*Statistical analysis.* GraphPad Prism 9 was used for graphic representation and statistical data analysis. We used a two-tailed unpaired Student’s *t*-test to compare two means with equal variance. Differences were considered statistically significant when *p* < 0.05.

## Results

### **A novel in vitro** **flow chamber system to study blood component and coronary scaffold interactions.**

To analyze the thrombogenicity of coronary scaffolds in vitro, scaffold material was embedded in commercially available µ-Slide I Luer channel mimicking a blood vessel lumen (Fig. 1A). To avoid scaffold strut collapse of high-pressure inflated 3 mm diameter scaffolds in the µ-Slide, scaffolds were cut in half along a pre-defined line to that prevents scaffold structure disintegration (Fig. 1B). With two rows of connectors per half scaffold remaining, scaffold stability was not compromised. To insert a half scaffold into the channel slide, two orientations were tested: (*i*) The open-side-up orientation and (*ii*) open-side-down with cut ends pointing upwards or downwards, respectively. Half scaffolds embedded in the open-side-down orientation induce microfractures of the sealing coverslip caused by the sharp end of the cutting edges (data not shown). In contrast, the open-side-up scaffold orientation was technically easy to mount (Fig. 1A). Furthermore, the open-side-up orientation of a half scaffold allowed for convenient imaging of the entire half scaffold within the range of the objective’s working distance. To allow for long-term exposure of blood components to the half scaffold under physiological conditions, the channel slide containing the half scaffold was subsequently placed in a conventional cell culture incubator (Fig. 1C).

### **BIOmag scaffold material shows strongly reduced** in vitro **thrombogenicity.**

In order to test for thrombogenic events on different stent and scaffold surface material, freshly isolated platelet-rich plasma (PRP) from buffy coats was perfused in flow chamber systems with Magmaris-316 L, Magmaris RMS and DREAMS 3G prototype devices. Devices were exposed to a laminar platelet flow of 10dyn/cm^2^ for 2 h. High-resolution immunofluorescence imaging of the total scaffold and stent strut areas revealed micro-thrombotic events on all half devices within the region of direct contact between the strut crown and the glass coverslip (Fig. 1D), which were likely caused by disturbed flow in that particular area, irrespective of the scaffold material’s nature (**Supplemental video 1**). This specific area was therefore excluded from consecutive analyses. Notably, along the strut length, where laminar-like flow was obtained (**Supplemental video 2**), platelet adhesion and coverage were strongly reduced on DREAMS 3G prototype and Magmaris RMS half scaffolds compared to the control Magmaris-316 L device (Fig. 1E).

**Figure 2:**
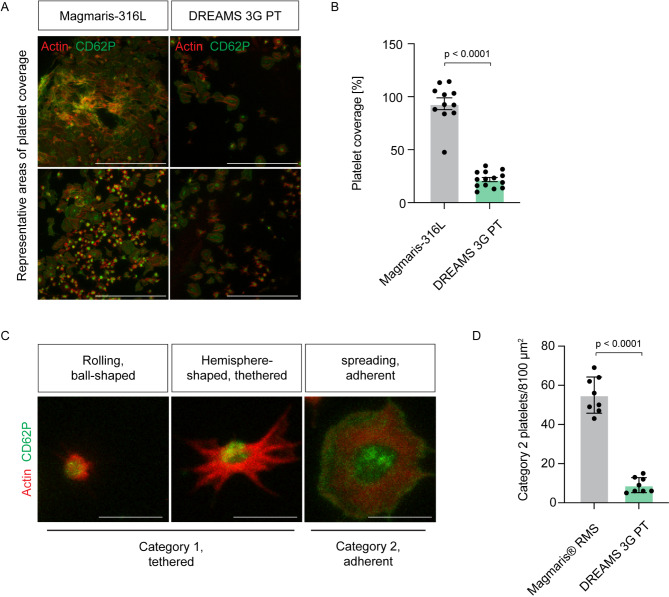
Thrombogenicity of the DREAMS 3G prototype made from BIOmag material is significantly diminished. (**A**) Individual flow experiments were conducted with PRP obtained from the same buffy coat for all scaffolds. Laminar shear stress of 10dyn/cm^2^ was applied for 2 h. Representative immunofluorescence of platelets attached to Magmaris-316 L and DREAMS 3G prototype (PT) scaffolds using an antibody against CD62P (green) and phalloidin (red, actin). (**B**) Quantification of platelet coverage (*n* = 3–6 images per scaffold from three independent experiments). Data represent mean ± s.e.m. p value, Two-tailed unpaired Student’s *t*-test. (**C**) Representative immunofluorescence of platelets attached to scaffold material in a reversible manner (category 1: rolling, ball-shaped and hemisphere-shaped) and in an irreversible manner (category 2: spreading platelet). (**D**) Irreversible platelet adhesion to the DREAMS 3G prototype scaffold is significantly diminished. Individual flow experiments were conducted with PRP obtained from the same buffy coat for all scaffolds. Laminar shear stress of 10dyn/cm^2^ was applied for 2 h. Quantification of irreversibly attached platelets (category 2) (*n* = 4 images per scaffold from two independent experiments). Data represent mean ± s.e.m. p value, Two-tailed unpaired Student’s *t*-test. Scale bars: 50 μm (**A**), 5 μm (**C**).

Next, we aimed to evaluate *in vitro* thrombogenicity of the novel DREAMS 3G prototype scaffold made of the BIOmag material, which is the next-generation development of its predecessor, the Magmaris RMS scaffold. We first compared platelet coverage of the DREAMS 3G prototype half scaffold to the backbone equivalent Magmaris-316 L. The devices were exposed to PRP samples from the same donor and exposed to laminar flow of 10dyn/cm^2^ for 2 h. We found that platelet coverage of DREAMS 3G prototype half scaffolds was significantly reduced by 77% when compared to Magmaris-316 L half device (Fig. 2A, B). Next, we sought to compare *in vitro *thrombogenicity of the DREAMS 3G prototype scaffold directly to its predecessor scaffold Magmaris RMS. Procoagulant surface-mediated platelet activation proceeds via rolling, initial tethering, firm adhesion and spreading^14^, Fig. 2C). We categorized platelet adhesion into category 1 (rolling and hemisphere-shaped) and category 2 (spreading) (Fig. 2C). To investigate scaffold-driven platelet activation we focused on firm adhesion and spreading events (category 2) to Magmaris RMS and DREAMS 3G prototype scaffolds and performed high-magnification immunofluorescence imaging. We found that firm adhesion and spreading of platelets to the BIOmag material of the DREAMS 3G scaffold was strongly reduced by 84% compared to the Magmaris RMS material (Fig. 2D), confirming its reduced thrombogenicity.

## Discussion

There is a demand for in vitro stent systems that can function as platforms to study scaffold and stent devices in a controlled environment. These systems provide valuable insights that complement in vivo studies, guiding the development of innovative devices. Simultaneously, they contribute to efforts aimed at minimizing animal experimentation in accordance with the principles of Replacement, Reduction, and Refinement (3R)^15^. Here, we established a novel and easy-to-use in vitro stent flow chamber system to study thrombogenicity of different stent and scaffold material. In a proof-of-concept study, our system revealed reduced thrombogenicity of the novel DREAMS 3G prototype exhibited strongly reduced *in vitro* thrombogenicity compared to its predecessor scaffold Magmaris RMS and the Magmaris-316 L stent backbone equivalent.

While contemporary drug eluting stents have a 2-year thrombosis rate of approximately 0.6-1%, RMS devices consistently showed very low 2-year scaffold thrombosis rates in clinical trials^16,17^. For example, improved clinical safety and reduced thrombogenicity of the Magmaris scaffold has been reported in a pooled analysis of BIOSOLVE-II and -III^7^with no scaffold thrombosis up to 36 months, and 0.8% (or 0.4% without early antiplatelet or anticoagulant interruption) scaffold thrombosis after 25 months in the BIOSOLVE -IV full cohort study^8^. Furthermore, the final DREAMS 3G design, tested in the BIOMAG-I study^18^, also showed 0% definite or probable scaffold thrombosis, confirming the low thrombogenic potential of magnesium based resorbable scaffolds in general.

A previous study using a porcine arteriovenous shunt model could further demonstrate that the Magmaris RMS exhibits less thrombogenicity and inflammatory cell deposition compared not only with the Magmaris-316 L backbone equivalent^10^but also compared to Orsiro, a contemporary ultrathin drug eluting stent with extensive clinical evidence^19,20^. Notably, our *in vitro* analysis reveals that the BIOmag material in the DREAMS 3G prototype significantly outperforms its predecessor, Magmaris RMS, by exhibiting reduced platelet coverage and spreading. Our findings are in line with a first-in-human study demonstrating that at the end of the resorption period the final DREAMS 3G device is clinically safe and effective^11^. Furthermore, the BIOmag material showed a significantly lower scaffold discontinuity when compared to Magmaris RMS material^9^, likely providing a more efficient and longer scaffolding time.

While the in vitro stent flow chamber system introduces fluid flow and shear stress, it lacks other mechanical forces of the physiological in vivo microenvironment such as extracellular matrix stiffness or stretch^21,22^. Micro-thrombotic events were observed in all half devices within the region of direct contact between the strut and the glass coverslip. This observation indicates that the occurrence of micro-thrombotic events in this area is artificially induced, resulting from disturbed flow, irrespective of the scaffold material’s nature. The in vitro stent flow chamber system therefore should only be utilized to investigate blood component interactions along the strut length, while avoiding areas of artificially perturbed flow. Future flow systems should implement a physiological vessel wall microenvironment, including hydrogel embedding and extracellular matrix components. This approach would also create a natural vessel-shaped anatomy around the devices.

Future applications employing the novel flow chamber system could include the study of device endothelialization^23,24^ and live imaging of blood component and endothelial cell interaction with device material. Furthermore, the *in vitro* stent flow chamber system has the potential to advance personalized medicine, e.g., by optimizing device material and drug elution tailored to patient needs or by analyzing and mitigating the individual’s risk of thrombosis and restenosis.

## Electronic supplementary material

Below is the link to the electronic supplementary material.

Supplementary Material 1

Supplementary Material 2

Supplementary Material 3

## Data Availability

All data is provided within the manuscript or supplementary information files. Raw data are available from the corresponding author upon request.
