# Supplementary material for: A novel stent flow chamber system demonstrates reduced thrombogenicity of bioresorbable magnesium scaffolds
Source: Sci Rep. 2024 Nov 4;14:26691. doi: 10.1038/s41598-024-77266-0 (PMC11535548; doi:10.1038/s41598-024-77266-0)
Supplement: Supplementary file 3 — Supplementary Material 3 [file 41598_2024_77266_MOESM3_ESM.docx]

**A novel stent flow chamber system demonstrates reduced thrombogenicity of bioresorbable magnesium scaffolds**

Müller *et al.*

**Supplemental videos**

**Supplemental video 1: Disturbed platelet flow within the region of contact between the strut crown and the glass coverslip.**

**Supplemental video 2: Along the strut length laminar-like platelet flow is maintained.**
